# A brief telehealth brain health program for family caregivers of persons living with dementia: the Brain Health for Me^©^ preliminary efficacy study

**DOI:** 10.3389/fpsyg.2026.1821545

**Published:** 2026-05-25

**Authors:** María P. Aranda, Jiaming Liang, Yvette I. Cordero

**Affiliations:** 1 Suzanne Dworak-Peck School of Social Work, University of Southern California, Los Angeles, CA, United States; 2 Edward R. Roybal Institute on Aging, University of Southern California, Los Angeles, CA, United States; 3 School of Public Health, University of Texas Health Science Center at Houston, Houston, TX, United States

**Keywords:** anxiety, brain health, dementia caregivers, efficacy, physical activity, social activity, stress management, technology

## Abstract

**Introduction:**

Family caregivers of persons living with dementia experience high stress, elevated rates of depression and anxiety, and an increased risk of cognitive decline compared to non-caregivers. Applications of telehealth interventions in the field of aging and geriatrics can overcome geographic and logistical barriers in the delivery of education and lifestyle support. We report preliminary efficacy findings for Brain Health for Me (BH4Me)^©^, a brief, co-designed telehealth intervention promoting dementia knowledge, healthy lifestyles, and wellbeing among dementia caregivers.

**Methods:**

This single-arm, pre-post study evaluated the preliminary efficacy of BH4Me^©^ with 59 racially- and ethnically-diverse dementia caregivers based on a three-session co-designed psychoeducational and skill-building program on dementia-knowledge and brain-health pillars: physical activity, social engagement, stress management, sleep, mental stimulation, healthy diet, chronic condition management, and substance use. Weekly session integrated brief lectures with guided discussion, Q&A, and interactive games to enhance engagement and skill application.

**Results:**

Participant reports showed significant improvements in physical activity, social engagement, stress-management, self-efficacy, negative relationship quality, and anxiety, highlighting the potential of telehealth lifestyle and psychoeducational approaches to enhance caregivers' brain health and emotional wellbeing. Delivering the program virtually overcame geographic and scheduling barriers and fostered group interaction that may have reinforced sustained behavior change.

**Discussion:**

Our study provides preliminary evidence of positive outcomes in the areas of lifestyle health behaviors, self-efficacy, and anxiety, thus contributing to the evidence on interventions targeting dementia caregivers. Future work should focus on larger randomized trials, longer observation periods, and objective, real-time measures to evaluate caregiver knowledge and skill acquisition.

## 1 Introduction

Dementia affects over seven million older adults in the United States and is projected to nearly double by 2050, placing immense demands on family caregivers who provide more than 80% of daily care and deliver over 18 billion hours of unpaid support each year (AARP and NAC, 2025; Alzheimer's Association, 2025). Persons living with dementia often struggle to maintain healthy routines, such as physical activity, diet, and sleep, accelerating their own cognitive and physical decline and heightening caregiver burden (Culberson et al., 2023). Caregivers of persons living with dementia (herein, dementia caregivers) experience high stress, financial strain, elevated rates of depression and anxiety, and an increased risk of cognitive decline (e.g., memory and executive function) compared with non-caregivers (Schulz et al., 2016; Stroud and Larson, 2021; AARP and NAC, 2025; Alzheimer's Association, 2025; Mudrazija and Aranda, 2025). For example, almost 60% of dementia caregivers report at least one modifiable risk factor that could increase their own chances of developing dementia (smoking, hypertension, poor sleep), and upwards of one out of four (24.3%) report multiple risk factors (Alzheimer's Association, 2024b).

Substantial evidence exists that dementia caregivers forego caring for their health in the form of missing doctor appointments, delaying routine health checkups, poor nutrition, sedentariness, low social engagement, poor sleep hygiene, and maladaptive stress management (Schulz et al., 2016; Stroud and Larson, 2021; AARP and NAC, 2025; Alzheimer's Association, 2025). While several evidenced-based dementia skill-building interventions exist, most focus primarily on caregiver burden, coping, or caregiving skills rather than directly targeting the caregiver's own brain health and modifiable lifestyle behaviors (Kishita et al., 2018). A large meta-analysis of caregiver interventions further suggests that psychoeducational and multicomponent programs can improve caregiver outcomes, although effects are generally modest, underscoring the need for innovative and more targeted approaches (Walter and Pinquart, 2020). In this context, a brain health framework offers added value by positioning caregiver self-care not only as a way to improve immediate wellbeing, but also as a strategy to support longer-term cognitive and overall health.

This need is also reflected in international literature, which has highlighted dementia caregiving as a broader psychosocial and societal challenge shaped by stigma, limited support, and the need for stronger family- and community-based responses (Giannouli, 2017). Support for dementia caregivers therefore requires not only disease-related information, but also practical resources that help sustain their own wellbeing and functioning. Telehealth interventions for dementia caregivers, some of which date back several decades, can help address these needs by reducing time, transportation, and logistical barriers to participation while extending access to education and support (Farran et al., 2017; Fernandez-Bueno et al., 2024; Lumini et al., 2025). This is especially important given the real-world barriers to participation reported by dementia caregivers and service organizations in qualitative studies and reviews (Gutiérrez et al., 2022; Liang and Aranda, 2023; Aranda et al., 2024).

Although definitions of brain health abound in the scientific and popular literature, we draw from the World Health Organization definition, “Brain health is the state of brain functioning across cognitive, sensory, social-emotional, behavioral and motor domains, allowing a person to realize their full potential over the life course, irrespective of the presence of absence of disorder” (World Health Organization, 2022). The definition implies that prevention and early intervention is necessary and may be championed by addressing different components or determinants of brain health, that is, cognitive stimulation, social engagement, lifestyle and health management practices, among others. In the last decade there has been a surge of brain health programs on a global scale dedicated to the general population in the forms of intensive, long-term brain health programs in the US and abroad. For example, the Finnish Geriatric Intervention Study to Prevent Cognitive Impairment and Disability (FINGER) study is the first randomized trial worldwide demonstrating that multidomain lifestyle interventions can improve brain health and prevent cognitive decline (Ngandu et al., 2015). Similarly, the US POINTER study found that the structured, higher-intensity intervention had a greater benefit on cognition than the low-intensity intervention (Baker et al., 2025). Although the studies did not specifically target dementia caregivers, the evidence for these large studies is hopeful. It is unknown if dementia caregivers would avail themselves of such programs due to the intensity and commitment required over the 2 years. Lack of awareness about said programs, time unavailability, need for dependent adult care, navigating technology, family dynamics, and overall personal costs related to participation (transportation, time off from work), etc. are real-world barriers that may interfere with such participation in multidomain lifestyle interventions (Ong et al., 2025; Sheffler et al., 2026).

In this study, we report the preliminary efficacy for Brain Health for Me (BH4Me)^©^, a brief co-designed telehealth program among dementia family caregivers on a variety of primary outcomes (healthy lifestyle behaviors) and secondary outcomes (self-efficacy, relationship quality, depressive, and anxiety symptoms). Establishing efficacy of community-based brain health interventions is a key step in informing large-scale studies to mitigate individual and societal burdens of dementia family caregiving.

## 2 Methods

### 2.1 Study overview

We adopted a single-arm, non-randomized pre-post study to test the preliminary efficacy of the BH4Me^©^ intervention among dementia family caregivers using a three-timepoint assessment: pre-intervention/baseline, post-intervention follow-up, and 3-month follow-up. The intervention included one dementia knowledge module and eight brain health pillars: physical activity, social engagement, stress management, sleep, cognitive stimulation, healthy diet, chronic condition management, and smoking and alcohol control. The intervention was delivered vis-à-vis the Zoom videoconferencing platform from April through July 2024 (Zoom Video Communications, 2026). All sessions were conducted in English, with plans for future adaptation into additional languages and cultural contexts to enhance accessibility and relevance. The study was approved by the XXX (anonymized for peer review) Institutional Review Board and adhered to established ethical standards for human subjects research (Kurihara et al., 2024).

### 2.2 Brief description of the BH4Me^©^ intervention

The Brain Health for Me (BH4Me)^©^ program is a telehealth psychoeducational and skill-building course grounded in adult learning theory and experiential learning principles (Taylor and Hamdy, 2013; Surr et al., 2017). The curriculum consists of 3 weekly 90-min sessions combining structured instruction with interactive learning and short take-home assignments. Content is organized around one dementia knowledge domain and eight brain health pillars. The dementia knowledge domain covered normal cognitive aging vs. dementia, basic dementia concepts, Alzheimer's disease risk factors, and common caregiving challenges. This content was informed by educational materials from authoritative sources (Dua et al., 2017; National Institute on Aging, 2020; Alzheimer's Association, 2025). Eight evidence-based brain health pillars included: physical activity, social engagement, stress management, sleep, cognitive stimulation, healthy diet, chronic condition management, and smoking and alcohol control. Each session follows a standardized protocol that includes warm-up and homework review, didactic instruction interspersed with discussion or activities, and a closing segment focused on reflection and goal setting.

**Figure 1:**
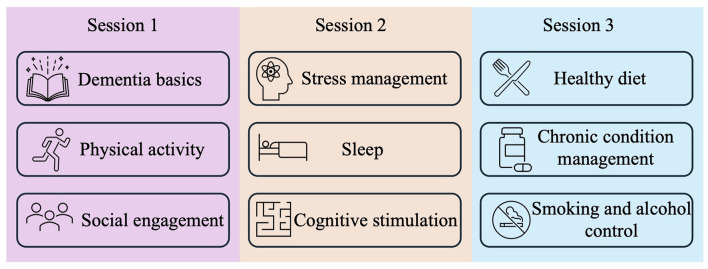
Overview of the BH4Me^©^ psychoeducational curriculum.

The intervention content was developed through a structured co-designed process that integrated empirical evidence with participant and provider expertise. The research team first conducted a comparative review of established dementia and brain health education programs and publicly available materials from leading health organizations to identify core behavioral targets and educational strategies relevant to dementia prevention and caregiver support (Harvard Medical School, 2000; National Institute on Aging, 2020; Alzheimer's Association, 2024a). These domains were then cross-validated against scientific literature on modifiable lifestyle factors associated with cognitive health (Livingston et al., 2020; Geraets et al., 2023), resulting in a framework consisting of one dementia knowledge domain and eight brain health pillars. To ensure relevance, clarity, and feasibility for telehealth delivery, an advisory panel of dementia family caregivers, researchers, a geriatric neurologist, community health and social service providers participated in iterative co-design sessions to refine session content, language, and materials. Their feedback shaped the final curriculum structure, fostered practical suggestions for acceptability, relevance, and usability across diverse caregiver groups. A schematic overview of session content is presented in Figure 1.

### 2.3 Participants and recruitment

Following finalization of the intervention, a single-arm study was conducted to evaluate its preliminary efficacy. As a single-arm preliminary efficacy study, the primary purpose was to assess acceptability, retention, and initial within-person outcome changes. Thus, the target sample size was selected to be appropriate for pilot behavioral intervention research and to generate effect estimates and implementation information needed for planning a future larger controlled trial.

Inclusion criteria were selected to match the telehealth delivery format and target population of interest, including (1) being adult (≥21 years) family caregivers of persons living with dementia, (2) English-speaking, and (3) able to participate via Zoom videoconferencing with reliable internet and device access. Caregivers were asked during contact calls about technology access and literacy, and all confirmed their ability to participate without difficulty. Study materials and procedures were conducted via online data management platforms and email communications.

Recruitment proceeded continuously as the project progressed, with participants enrolled into multiple small cohorts as outreach activities were conducted. Outreach included educational booths, onsite and virtual presentations, and distribution of brain health and caregiver materials through 23 community events across XXX (anonymized for peer review) between April and July 2024.

**Figure 2:**
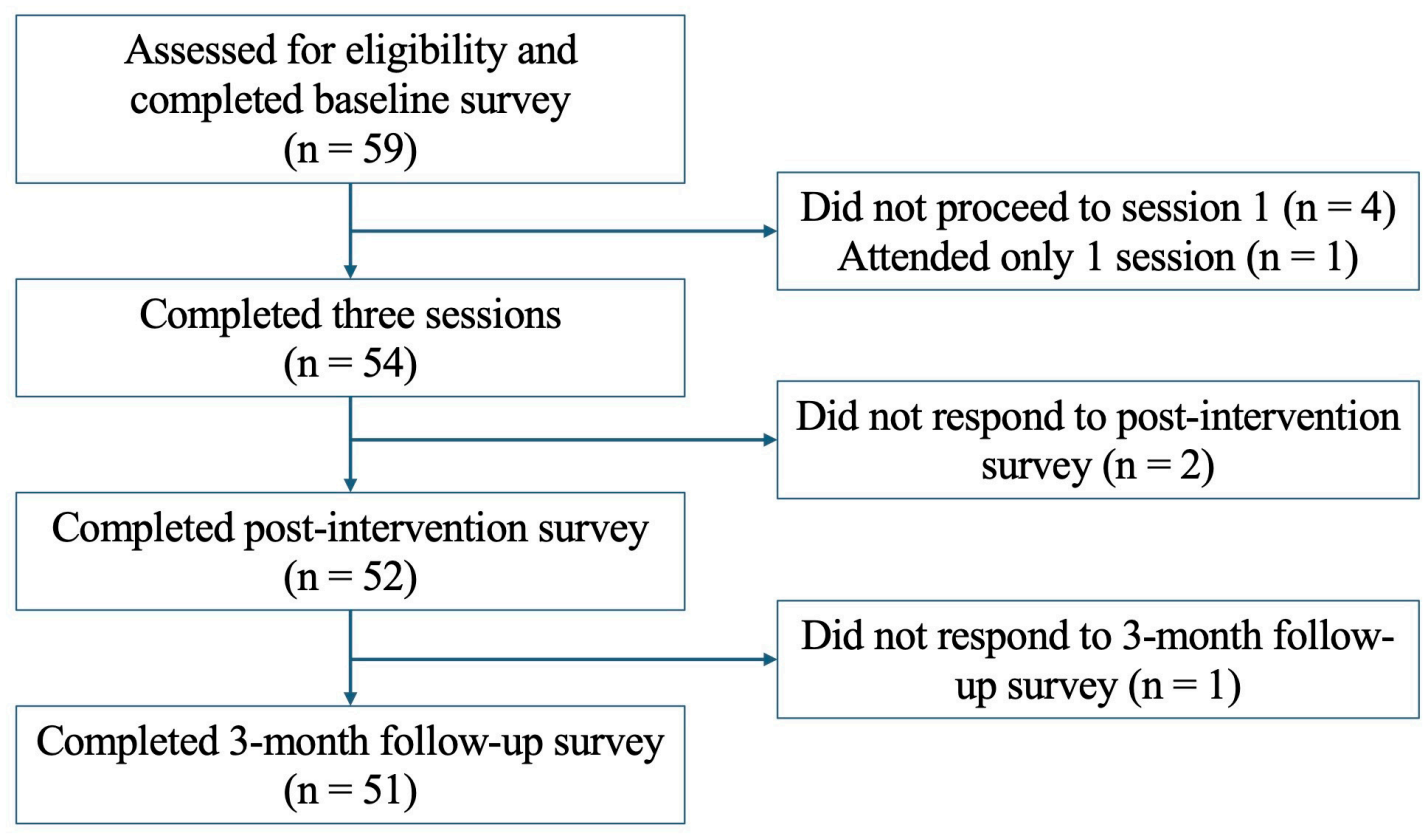
Enrollment, participation, and retention in the BH4Me^©^ pilot study.

In total, 59 caregivers completed the baseline survey, 52 (88.1%) completed all three sessions and post-intervention survey, and 51 completed the 3-month follow-up survey, indicating an 86% participant retention rate through the course of the study. Detailed enrollment and retention data are presented in Figure 2.

### 2.4 Intervention delivery

The intervention was delivered online via Zoom in small groups of fewer than 10 participants. Between April and July 2024, two to three cohorts were launched monthly, resulting in a total of 10 cohorts. All sessions were facilitated by the first two authors who also oversaw program quality and standardization. Both authors viewed session videotapes and provided continuous consultation on intervention development and testing, alignment with participant values and preferences. The third author provided participant recruitment, enrollment, and ongoing study navigation procedures to ensure engagement and troubleshooting between sessions. To maintain fidelity, each cohort followed an identical schedule of 3 weekly 90-min sessions based on uniform PowerPoint slides, digital handouts, self-assessment tools, and take-home assignments. Standardized intervention materials also included session-specific facilitator guidance notes, participant worksheets, and home-practice assignments aligned with each brain health topic; Figure 1 provides an overview of the session content and curriculum structure, and core intervention materials are available from the authors upon reasonable request. These materials were distributed after each session to reinforce key concepts and support behavior change. In addition to session-specific materials, caregivers received information on community-based health and social services to address concerns related to their own cognitive health and caregiving stressors.

### 2.5 Measures

Study outcomes were assessed at multiple timepoints corresponding to the program schedule. The baseline survey collected information on caregivers' sociodemographic characteristics (e.g., age, gender, education, income, marital status, employment, and ethnicity), health status, and caregiving-related factors, including relationship to the person living with dementia, duration and intensity of caregiving, co-residence, and the family member's functional limitations, assessed using caregiver-reported items on six activities of daily living (eating, toileting, bathing, dressing, grooming, and transferring) and seven instrumental activities of daily living (light chores, heavy chores, preparing meals, managing money, shopping, food shopping, and telephone use). For each activity, caregivers indicated whether the person could perform the task independently, with some help, or was fully dependent on others. We also collected program completion rates (surveys, class attendance), and Program Participation Questionnaire (PPQ) ratings for acceptability, convenience/usability, content quality, program structure, facilitator performance, general satisfaction, program recommendation, and privacy concerns (Berg et al., 2020). Pre-post class polls were implemented to identify immediate knowledge gains at the end of each session.

#### 2.5.1 Primary outcomes

*Dementia Knowledge Assessment Scale (DKAS)*. Knowledge about dementia was measured at baseline and post-intervention using the 25-item DKAS (Annear et al., 2017). The scale assesses understanding across dementia causes, care needs, and communication strategies. Items are rated on True (1) or False (0), with higher total scores indicating greater knowledge. The DKAS has demonstrated strong internal consistency and construct validity in caregiver and community samples. In this study, total scores were summed to reflect overall dementia knowledge at each timepoint.

*Caregivers' engagement and change in eight brain health related lifestyle domains* were assessed using a customized questionnaire that aligned with the program's eight brain health pillars: physical activity, social engagement, stress management, sleep, cognitive stimulation, healthy diet, chronic condition management, and substance use (smoking and alcohol). Several items were adapted from publicly available national health surveys and validated instruments.

At baseline, participants reported their current lifestyle patterns during the past month. Physical activity was measured by weekly frequency (days/week) and duration (hours/day). Social engagement, stress management, and cognitive stimulation activities were recorded as number of times per week. Baseline sleep practices were evaluated through questions on typical sleep duration, reasons for inadequate sleep, and specific sleep improvement behaviors (e.g., maintaining a schedule, creating a restful environment; Mastin et al., 2006). Dietary behaviors were captured by weekly frequency of fruit and vegetable intake (CDC, 2020). Chronic condition management was assessed through multiple-choice items (e.g., regular medical check-ups, medication adherence, self-monitoring). Tobacco use (not at all, some days, every day) and alcohol consumption (times in past 30 days) were queried using frequency questions consistent with public health surveillance standards (CDC, 2022).

For the post-intervention assessment, participants were asked to share practices or changes made since attending the program. Participants indicated whether they had initiated or increased practices across each of the eight brain health pillars (e.g., starting or increasing physical activity, adopting stress management techniques, improving sleep routines, increasing fruit/vegetable intake, planning regular check-ups, reducing smoking or alcohol use) as well as number of hours or frequency of such At the 3-month follow-up, the same domains were assessed for the “past 30 days.” It is important to note that although we asked about sleep duration and interruptions at baseline, we did not collect the same information at the follow-up assessments; we did ask a follow-up question to ascertain if they adopted healthy sleep practices as a result of taking the classes (yes/no).

#### 2.5.2 Secondary outcomes

*Revised Scale of Caregiver Self-Efficacy (RSCSE)* assessed the caregiver's perceived confidence in managing care-related challenges (Steffen et al., 2002). This 15-item instrument measures three subdomains: (1) obtaining respite, (2) responding to disruptive behaviors, and (3) controlling upsetting thoughts. Each item is rated from 0 (“not at all confident”) to 100 (“completely confident”), with higher scores indicating greater self-efficacy. The scale has demonstrated excellent internal consistency (Cronbach's α > 0.90) and construct validity among family caregivers of persons living with dementia (Steffen et al., 2019). Composite and subscale means were calculated for analysis.

*Relationship Quality Scale*, adapted from the National Study of Caregiving (NSOC), measured the caregiver's perceived relationship quality with the person living with dementia (Wolff et al., 2016). This measure includes separate subscales for positive relationship quality (e.g., affection, feeling appreciated) and negative relationship quality (e.g., criticism, tension). Items are rated on a four-point Likert scale (1 = “not at all” to 4 = “a lot”), with higher scores representing stronger endorsement within each dimension.

*Symptoms of depression and anxiety* were assessed using the Patient Health Questionnaire-2 (PHQ-2) and the Generalized Anxiety Disorder-2 (GAD-2; Kroenke et al., 2003; Spitzer et al., 2006). Each screener includes two items rated from 1 (“not at all”) to 4 (“nearly every day”), producing subscale totals from 2 to 8, with higher scores indicating more severe symptoms. Both measures have been validated in community and caregiver populations as brief, reliable indicators of emotional distress.

In addition to reporting their own behaviors, caregivers were asked at each follow-up assessment whether they assisted the person living with dementia with activities across the eight brain health pillars. Caregivers who responded “yes” selected the specific domains from a checklist that included items such as physical exercise, social interactions, stress management activities, cognitive stimulation, healthy diet, chronic condition management, and control of smoking and alcohol use. The total number of domains in which caregivers provided support was summed (range: 0–8), reflecting the breadth of lifestyle-related assistance offered to the person living with dementia.

### 2.6 Data analysis

Descriptive statistics (means, standard deviations, and percentages) were used to summarize the study participants' sociodemographic and caregiving related characteristics. For continuous outcomes measured across multiple timepoints, linear mixed-effects models (LMMs) were estimated to examine within-subject changes over time while accounting for individual variability. Time (baseline, post-intervention, and 3-month follow-up) was modeled as a categorical fixed effect, and participant as a random intercept, allowing for incomplete data at follow-up. Missing data were handled using full-information maximum likelihood (FIML) estimation, which provides unbiased parameter estimates under the assumption of missing at random (Tang and Tong, 2025). Given the preliminary nature and small sample size of the study, effect size estimates (e.g., Cohen's d) were not calculated. Analyses were conducted in Stata SE 18.0 (StataCorp LLC, 2023).

## 3 Results

### 3.1 Participant characteristics

Table 1 presents the descriptive characteristics. Among the 59 family caregivers who completed the baseline survey, the mean age was 57.68 years (SD = 12.27; range = 26–86), and the majority were women (81.36%). More than half were married or partnered (57.63%), 69.49% had a bachelor's degree or higher, and 70.45% reported annual household incomes above USD 50,000.

**Table 1:** Descriptive characteristics of family caregivers (*N* = 59).

Variable	*N* (%)	Mean (SD) [range]
Age (years)		57.68 (12.27) [26, 86]
Sex (female)	48 (81.36%)	
Marital status (married/partnered)	34 (57.63%)	
Education (bachelor's degree or above)	41 (69.49%)	
Annual household income (>50K USD)	31 (70.45%)	
Race/ethnicity		
Non-Hispanic White	15 (25.42%)	
Non-Hispanic Black	15 (25.42%)	
Hispanic	19 (32.20%)	
Other race/ethnicity	10 (16.95%)	
Relationship type		
Spouse	37 (62.71%)	
Adult child	14 (23.73%)	
Other	8 (13.56%)	
Years of being caregiver		4.56 (3.68) [0.4, 17.6]
Hours of caregiving per week (excluding 24/7 caregivers)		34.83 (23.24) [4, 80]
Number of caregivers reported 24/7 caregiving	16 (27.12%)	
Having dementia diagnosis	50 (84.75%)	
Subjective memory complaint by caregiver	23 (28.98%)	

The caregivers were racially and ethnically diverse: 25.42% identified as non-Hispanic White, 25.42% as non-Hispanic Black, 32.20% as Hispanic, and 16.95% as other race or ethnicity (including Chinese, Vietnamese, Filipino, and Thai). Most caregivers were spouses (62.71%) or adult children (23.73%) of the person living with dementia. Caregivers had been providing care for an average of 4.56 years (SD = 3.68). Among those not reporting continuous (24/7) caregiving, the mean caregiving intensity was 34.83 hours per week (SD = 23.24). Sixteen caregivers (27%) reported providing care on a 24/7 basis. Most indicated that the care recipient had a formal dementia diagnosis (84.75%), and 28.98% of caregivers endorsed experiencing subjective memory complaints themselves.

### 3.2 Participation and acceptability

**Figure 3:**
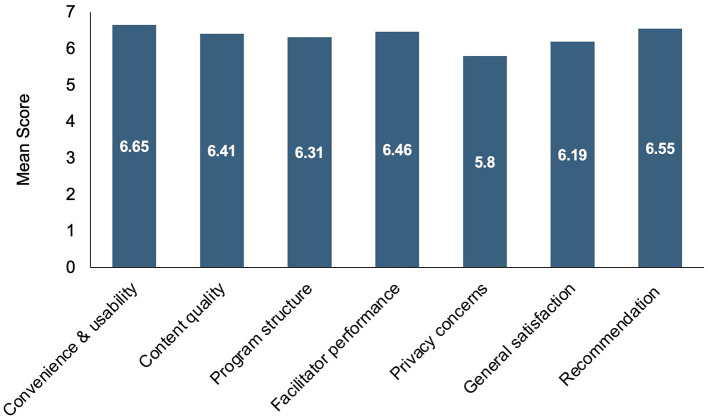
Mean Program Participation Questionnaire (PPQ) scores.

Of the 59 caregivers who completed baseline survey, 52 (88.1%) attended all three BH4Me^©^ sessions and 51 (86.4%) completed the 3-month follow-up. Program Participation Questionnaire ratings indicated strong acceptability on a seven-point scale: convenience/usability (*M* = 6.65, SD = 0.62), content quality (*M* = 6.41, SD = 0.87), program structure (*M* = 6.31, SD = 0.85), facilitator performance (*M* = 6.46, SD = 0.67), general satisfaction (*M* = 6.19, SD = 0.96), program recommendation (*M* = 6.55, *SD*=0.78), and privacy concerns (*M* = 5.80, SD = 1.81; see Figure 3). Pre-post class polls, which were used as brief session-specific formative checks rather than standardized study outcomes, showed immediate knowledge gains within each session (data not shown and available from the authors upon request), further supporting the program's feasibility and perceived value.

### 3.3 Primary outcomes

Table 2 summarizes changes in dementia knowledge and engagement in the eight brain health lifestyle domains across baseline, post-intervention, and 3-month follow-up. Although there was an upward trend in caregiver knowledge of dementia from baseline, the increase was not significant (*M* = 12.03, SD = 2.20) to post-intervention (*M* = 12.56, SD = 2.04).

**Table 2:** Primary outcomes (dementia knowledge and eight brain health pillars).

Variable		% reported changes, practices	Baseline	Post-intervention	3-month follow-up
			Mean (SD) [range]/[*N* (%)]		
Dementia knowledge score			12.03 (2.20) [10, 17]	12.56 (2.04) [10, 19]	
Physical Exercise (hours/week)		62.75%	3.92 (5.72) [0, 28]	6.13 (5.39) [0, 21]^**^	5.14 (5.34) [0, 25]
Social activity (times/week)		33.33%	2.98 (2.35) [0, 10]	3.06 (2.30) [0, 10]	3.83 (2.60) [0, 10]^*^
Stress management practice (times/week)		31.37%	2.95 (2.49) [0, 10]	2.90 (2.51) [0, 10]	4.19 (2.83) [0, 10]^**^
	Perceived stress	/	1.77 (1.22) [0, 4]	1.47 (1.10) [0, 4]	1.54 (1.25) [0, 4]
Sleep quality practice		43.14%	/	/	/
Cognitive stimulation (times/week)		37.25%	4.40 (2.95) [0, 10]	4.27 (2.58) [0, 10]	4.82 (2.93) [0, 10]
Healthy Diet	Fruit intake (times/week)	23.53%	4.78 (2.17) [0, 7]	4.94 (2.13) [0, 7]	4.93 (2.32) [0, 7]
	Vegetable intake (times/week)	31.37%	5.09 (1.78) [0, 7]	4.83 (1.75) [2, 7]	5.23 (1.99) [0, 7]
No. of chronic management practices		42.00%	1.85 (1.62) [0, 5]	1.92 (1.24) [1, 5]	2.22 (1.43) [1, 5]
Substance use	Cigarette use	/	[3 (3.57%)]	/	/
	Alcohol use	11.76%	1.63 (2.31) [0, 8]	1.43 (2.11) [0, 8]	1.86 (2.58) [0, 8]

^*^p < 0.05, ^**^p < 0.01.

**Figure 4:**
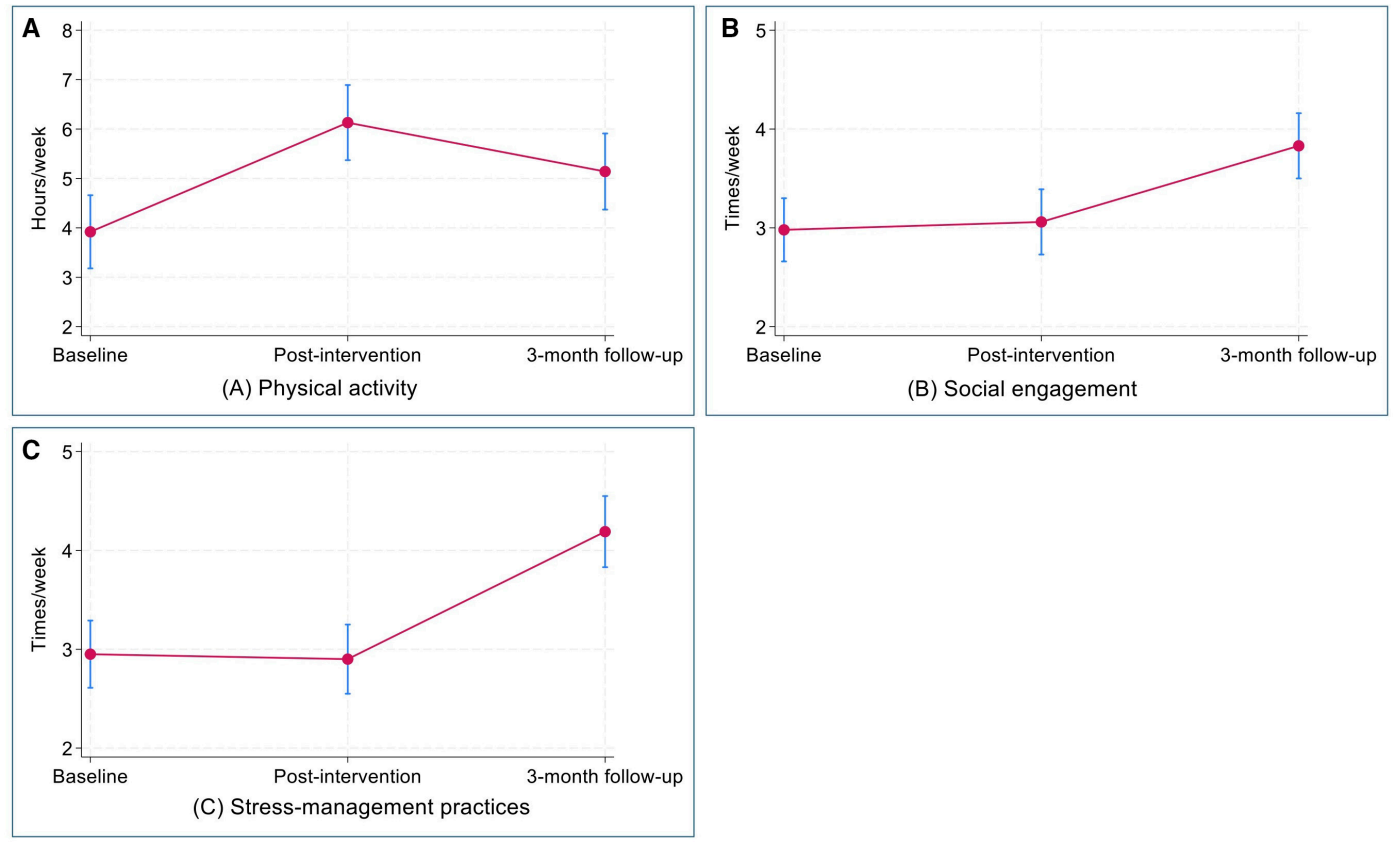
Changes in key brain health lifestyle behaviors across baseline, post-intervention, and 3-month follow-up. **(A–C)** display mean levels and standard error bars for **(A)** physical exercise (hours/week), **(B)** social engagement (times/week), and **(C)** stress management practices (times/week) across the three assessment timepoints.

Significant improvements were observed in three lifestyle behaviors targeted by the BH4Me^©^ program. Physical activity increased from 3.92 h/week (SD = 5.72) at baseline to 6.13 h/week (SD = 5.39) post-intervention (*p* < 0.01), with a slight decline at the 3-month follow-up (5.14 h/week, SD = 5.34). Social engagement remained stable immediately after the program but increased significantly by follow-up (2.98–3.83 times/week, *p* < 0.05). Stress management practices followed a similar pattern, increasing significantly from baseline to the 3-month follow-up (2.95 to 4.19 times/week, *p* < 0.01). Figure 4 displays these trajectories for physical exercise (Figure 4A), social engagement (Figure 4B), and stress management practices (Figure 4C).

Engagement in other brain health domains remained generally stable over time. Cognitive stimulation activities showed minimal variation from baseline to follow-up. Fruit and vegetable intake also remained largely unchanged, with mean weekly servings showing only small fluctuations. Chronic condition management behaviors increased modestly across the three timepoints (from 1.85 to 2.22 strategies endorsed), although these changes did not reach statistical significance in the mixed-effects models.

For baseline sleep related behaviors, approximately only one-third of caregivers (32%) reported achieving the recommended 7–9 h of sleep per night, and 42% indicated that caregiving responsibilities negatively affected their sleep quality. Caregivers also identified multiple reasons for inadequate sleep, including stress, nighttime caregiving demands, and other health related conditions. Following the intervention, 43% of participants reported adopting at least one new sleep improvement practice (e.g., maintaining a consistent schedule or creating a restful sleep environment).

Regarding cigarette use and alcohol drinking, at baseline, only three caregivers (3.57%) reported current cigarette smoking, and no meaningful changes were observed over time. Alcohol consumption levels were low overall, averaging fewer than two drinking occasions in the past 30 days at each assessment. Although 11.76% of participants reported making changes to alcohol use following the intervention, mean drinking frequency did not significantly change across timepoints.

### 3.4 Secondary outcomes

Table 3 presents changes in caregiver self-efficacy, relationship quality with the person living with dementia, emotional symptoms, and the number of brain health lifestyle domains in which caregivers provided support. Caregiver self-efficacy improved significantly from baseline (*M* = 68.41, SD = 19.71) to post-intervention (*M* = 74.33, SD = 17.24; *p* < 0.05), with modest reduction at the 3-month follow-up (*M* = 72.29, SD = 16.93). Improvements were driven largely by the “obtaining respite” domain, which increased from 52.81 to 64.88 (*p* < 0.05), and the “controlling upsetting thoughts” domain, which increased from 76.68 to 81.36 (*p* < 0.05). The “responding to disruptive behaviors” subscale showed minimal change across time.

**Table 3:** Secondary outcomes.

Variable	Baseline	Post-intervention	3-month follow-up
	Mean (SD) [range]		
Caregiver self-efficacy (total)	68.41 (19.71) [23, 99]	74.33 (17.24) [29, 100]^*^	72.29 (16.93) [37, 100]
Obtain respite	52.81 (38.14) [0, 100]	64.88 (31.68) [0, 100]^*^	57.39 (35.29) [0, 100]
Respond to disruptive behaviors	79.04 (21.34) [20, 100]	78.69 (20.53) [18, 100]	80.99 (22.06) [0, 100]
Upset thoughts control	76.68 (19.52) [13, 100]	81.36 (16.74) [14, 100]^*^	80.23 (16.72) [24, 100]
No. of helped healthy lifestyles	3.37 (1.99) [0, 8]	3.40 (2.43) [0, 8]	3.31 (2.01) [0, 8]
Relationship quality			
Positive	6.71 (1.48) [3, 8]	6.78 (1.51) [4, 8]	6.74 (1.47) [3, 8]
Negative	5.34 (1.49) [2, 8]	4.02 (1.95) [2, 8]^***^	4.59 (1.60) [2, 8]^***^
Mental health			
Depression (PHQ-2)	3.50 (1.35) [2, 7]	3.23 (1.21) [2, 6]	3.29 (1.55) [2, 8]
Anxiety (GAD-2)	3.98 (1.29) [2, 8]	3.57 (1.25) [2, 6]^*^	3.76 (1.44) [2, 8]

^*^p < 0.05, ^***^p < 0.001.

Relationship quality with the person living with dementia also showed favorable patterns. Negative relationship quality decreased significantly from baseline (*M* = 5.34, SD = 1.49) to post-intervention (*M* = 4.02, SD = 1.95; *p* < 0.001) and remained lower at follow-up (*M* = 4.59, SD = 1.60; *p* < 0.01), indicating reduced relational strain. Positive relationship quality remained stable.

Mental health indicators showed selective improvements. Anxiety symptoms (GAD-2) decreased significantly from baseline (*M* = 3.98, SD = 1.29) to post-intervention (*M* = 3.57, SD = 1.25; *p* < 0.05), with slight increase at follow-up (*M* = 3.76, SD = 1.44). Depressive symptoms (PHQ-2) changed minimally.

Caregivers' reports of helping the person living with dementia engage in brain healthy lifestyles remained stable across time (3.37 to 3.40 to 3.31 domains), suggesting no short-term change in caregiver-assisted behaviors.

## 4 Discussion

Brain health is a clinical and public health priority worldwide, and a growing area of concern for the millions of dementia family caregivers who put their own physical and cognitive health at risk to fulfill their family obligations and commitments (Schulz et al., 2016; Stroud and Larson, 2021; Alzheimer's Association, 2024b, 2025; AARP and NAC, 2025). This is the first study to test the preliminary efficacy of a brief online brain health intervention developed with–and for–diverse family caregivers of persons living with dementia. Unlike broader multicomponent caregiver programs that primarily emphasize burden reduction, coping, or caregiving skills, BH4Me^©^ explicitly organized its content around modifiable brain health domains that may support caregivers' longer-term cognitive and overall health in addition to immediate wellbeing. Our study provides preliminary evidence of positive outcomes in the areas of lifestyle health behaviors, self-efficacy, and anxiety, thus contributing to the evidence on interventions targeting dementia caregivers who are the backbone of health and long-term care services.

Our sample was comprised mostly of middle-aged women who were either spouses or adult children of the person they cared for—findings corroborated in the extant caregiver literature (Schulz et al., 2016; AARP and NAC, 2025; Alzheimer's Association, 2025). They were on average in the caregiving role for almost 5 years. Reflective of the geographical region from where they were selected, the sample was comprised of racially and ethnically diverse English-speakers which we attribute to our intensive, ongoing, and targeted recruitment approaches that are community engaged (NExTRAC, 2025). What was not expected was that almost 30% reported subjective memory complaints, a potential indicator of an unmet health need which we addressed by providing referrals to health and social services providers in the community. Although we did not examine predictors of these complaints in the present pilot study, they may reflect a combination of caregiver stress, mental health burden, sleep disturbance, midlife health risks, or age-related concerns that warrant further investigation in larger samples (Kinzer and Suhr, 2016).

Our study engagement and satisfaction indicators show that we had a high participant retention rate with 88% attending all three classes, and 86% remaining in the study through the last assessment. Second, participants consistently reported high satisfaction levels across all eight user-friendly domains such as acceptability: acceptability, convenience/usability, content quality, program structure, facilitator performance, general satisfaction, program recommendation, and privacy concerns. Data from exit interviews (data analysis in progress) indicate that although this type of information and format was new to the participants, they felt they learned a lot, understood the need to apply newly acquired skills and guidance, and were eager to continue said skills. One area of concern was the brevity of the intervention (“wanting more time to digest the information”) and wish to remain connected socially to the other peer participants (“like a support group”).

Turning to the study's primary outcomes, we found significant improvements for three of the eight brain health domains: social engagement activities, stress management practices, and physical activity. While remaining relatively flat at the post-intervention observation, both social engagement and stress management increased significantly at the 3-month follow-up, which indicates a slower but steady improvement trajectory over time. Physical activity increased after the intervention and although it decreased at the 3-month follow-up it remained higher than the baseline average.

With regards to the secondary outcomes, our findings indicate that psychosocial outcomes such as caregiver self-efficacy, and the quality of relationship with the person they care for changed significantly. For example, self-efficacy increased significantly at post intervention signaling a favorable trend toward caregivers perceiving that they have agency in changing their situation. It is notable that the strongest drivers of self-efficacy improvements were in two specific areas: the ability of the caregiver to obtain respite; and to control upsetting thoughts—two potentially malleable skill-building strategies and areas to target interventions. Prior evidence from the field of cognitive psychology indicates that self-efficacy is a potential mechanism of action or pathway to change in individual health behaviors, and specifically dementia caregivers (Mowat and Laschinger, 1994; Bandura, 1997; Khan et al., 2021). Although we did not conduct mediational analysis, the role of self-efficacy in mediating change in caregiver's health-related behaviors should be explored in future efforts.

Our study tested if the quality of the relationship between caregiver and the person they cared for changed by examining both positive and negative aspects of the relationship given that relationships typically demonstrate a valence of positive and negative aspects (Cantu and Aranda, 2023)—reflecting real-world family dynamics. Positive relationship quality remained stable, while negative relationship quality decreased significantly indicating reduced relational strain. In fact, of all the outcomes in our study, negative relationship quality declined steadily throughout the entire observation period. Reasons for this remain unknown, but we conjecture that information from the classes, practice assignments, and emotional support from other participants (sharing personal narratives) could have mitigated the conflictual components of care that sometimes emerge in caregiving. Additionally, greater dementia knowledge and improved healthier practices may have helped some caregivers interpret the family members' neuropsychiatric behaviors or symptoms with less reactivity or confrontation (Isik et al., 2019; Meyer et al., 2023). Lastly, the self-care elements of the intervention could have imbued a more positive light on the caregiving situation, thus letting go of negative cognitions regarding the relationship. Previous work examining relationship quality among caregivers indicate that relationship quality is related to neuropsychiatric symptoms, specifically behavioral problems that may be stigmatized and unpredictable (Cantu and Aranda, 2023). Relationship quality and managing neuropsychiatric symptoms of dementia can be addressed in brain health interventions due to their malleable nature and potential intervention focus in the stress management module, and discussions around reaching out for medical advice (Sanosi et al., 2025).

Mental health indicators showed improvement for anxiety symptoms which decreased significantly at post intervention, with a slight uptick at the 3-month follow-up. The depressive symptom outcome showed no improvement. We can extrapolate several notions for the nonsignificant finding for depressive symptoms: The average baseline scores indicated low depressive symptom rates; the intervention was too brief; and/or the measure did not capture the full extent of affective symptomatology (only the two-symptom screener was used). Previous work on dementia caregiver depression interventions have shown that multimodal assessments and interventions are more effective (Huang, 2022). Future interventions may also consider incorporating spirituality- or meaning-centered components, as emerging evidence suggests that such approaches may provide additional psychosocial support for some dementia caregivers (Giannouli and Giannoulis, 2020). Future work should also take a careful look at the mechanisms of intervention design that realistically can change depressive symptoms over time, and within longer observation intervals.

Turning to the technological aspects of our study, caregivers were relatively fluid in navigating with online study procedures (consent forms, questionnaires), class session videoconferencing, email correspondence, etc. No more than 5% of the participants required assistance from research personnel with digital technology. This could be attributed to the fact that about 70% had a bachelor's degree or higher. How such technological literacy is navigated with participants with lower levels of education and exposure to such technology remains unknown although we suspect that more navigation and engagement tasks would be expected from research personnel.

As stated by Huang (2022), technology-driven models could provide a handy and lower cost intervention compared to traditional home care, supply a family caregiver with social interaction and emotional support and facilitate information exchange with other caregivers and professionals. It could also ameliorate the decision-making process for matters about patient care. The literature also suggests that technology-based interventions can reduce caregiver burden and stress and may improve caregiver self-efficacy and quality of life, although effects vary across intervention types and study designs (Fernandez-Bueno et al., 2024; Lumini et al., 2025).

## 5 Limitations

The study has some limitations. First, it drew from a relatively small sample of almost 60 participants, and as a preliminary efficacy study, it was not intended to provide a definitive test of intervention effects. Second, although retention through follow-up was generally strong, several participants withdrew before completing all sessions or follow-up assessments. In a caregiver population with substantial time demands and fluctuating care responsibilities, such attrition is not unexpected, but it may still introduce bias and should be considered when interpreting these preliminary findings. Third, members of the study team who facilitated the intervention were also involved in program evaluation, which may have introduced some potential for response bias in self-reported outcomes, although the use of standardized quantitative measures helps reduce, though not eliminate, this concern. Fourth, because the intervention was delivered fully online, participation also required a basic level of technology access and literacy, which may have excluded or discouraged some caregivers with lower digital readiness and may limit generalizability to less technologically connected populations. Fifth, the brevity of the intervention is another limitation. Although the three-session format may have supported participation and retention (Barrio et al., 2025), some participants expressed a desire for more time, suggesting that the program may have been sufficient to raise awareness of needed skills and behavior changes, but less able to support their sustained practice and mastery. Finally, we did not ask the same sleep measures at baseline and follow-up, thus future studies should address sleep hygiene practices as shown in our prior work based on national surveys that dementia caregivers report elevated levels of sleep disturbance (Liang et al., 2020).

## 6 Conclusion

Our study provides preliminary evidence of positive outcomes in the areas of lifestyle health behaviors, self-efficacy, and anxiety, thus contributing to the evidence on interventions targeting dementia caregivers' brain health. Future work should focus on larger randomized trials, longer observation periods, and objective performance indicators (accelerometers, video observations) evaluate caregiver knowledge and skill application in real time (ecological momentary assessments, daily diaries). Subgroup analyses can provide needed information to ascertain who benefits the most from such brief online interventions, and their long-term effects. Lastly, a key health equity consideration is understanding that digital health interventions may systematically exclude caregiver populations—that for any one or a myriad of reasons—do not have the capacity, access, or tolerability of said interventions even at the time our world is experiencing gargantuan advances in digital health.

## Data Availability

Generic study materials are available upon request to interested researchers on a case-by-case basis. Raw data cannot be shared publicly or with third parties because doing so would risk violating the agreement signed by participants.
